# Primary cardiac lymphoma presenting with wide QRS tachycardia: a case report

**DOI:** 10.1093/ehjcr/ytaf065

**Published:** 2025-03-11

**Authors:** Ömer Şahin, Halime Tanriverdi, Turgut Seber, Arzu Taşdemir

**Affiliations:** Department of Cardiology, University of Health Sciences, Kayseri City Training and Research Hospital, 38080 Kayseri, Turkey; Department of Cardiology, University of Health Sciences, Kayseri City Training and Research Hospital, 38080 Kayseri, Turkey; Department of Radiology, University of Health Sciences, Kayseri City Training and Research Hospital, 38080 Kayseri, Turkey; Department of Pathology, Kayseri City Training and Research Hospital, 38080 Kayseri, Turkey

**Keywords:** Cardiac lymphoma, Wide QRS tachycardia, Cardiac MRI, Case report

## Abstract

**Background:**

Lymphomas, which originate from the haematopoietic system, are seldom found in the heart due to the absence of a lymphoid system. Primary cardiac lymphoma is quite rare. Cardiac lymphomas can present with dyspnoea, heart failure, pericardial effusion, and arrhythmia. Given the high mortality rates associated with cardiac masses, swift diagnosis is crucial.

**Case summary:**

A 46-year-old male patient presented to the emergency department of our hospital with complaints of dyspnoea and palpitations. The patient’s electrocardiogram revealed a tachycardia characterized by a wide QRS complex and a heart rate of 234 beats per minute, and an intravenous infusion of amiodarone was immediately started. In the cardiac MRI performed, a mass lesion was observed, which was ∼63 ∗ 30 ∗ 79 mm in size, extending from the right atrium to the superior vena cava and right atrial appendage, infiltrating the free wall of the right ventricle, pericardium, and right atrial wall, and showing distinct diffusion restriction in places. The patient’s cardiac MRI was documented with a suspicion for cardiac lymphoma. The patient was referred to haematology clinic and started on rituximab-cyclophosphamide, doxorubicin, vincristine, and prednisone chemotherapy.

**Discussion:**

The diagnosis of primary cardiac lymphoma is uncommon. Even a mass exceeding 7 cm in size may not be visible on transthoracic echocardiography. The use of cardiac MRI to identify intracardiac masses should be incorporated into the diagnostic process to expedite diagnosis and the initiation of life-saving treatment.

**Learning points**

- A mass exceeding 7 cm may not be observed on conventional imaging methods as in our case. So, we aimed to raise awareness about this disease and emphasize the importance of multimodality imaging methods in early diagnosis.
- Although primary cardiac lymphoma is a challenging clinical condition with non-specific symptoms, it may present with atrial tachycardia with aberrant conduction, as observed in our case. This illustrates the importance of maintaining a high level of suspicion to facilitate the diagnosis.

## Introduction

Though cardiac tumours are infrequent, the majority are of metastatic origin when compared to primary cardiac tumours. The most seen metastatic tumours include lung cancers, specifically pleural mesothelioma, adenocarcinoma, and squamous cell carcinoma. Additionally, malignant melanoma and breast cancer are prominent in this category.^1^ Lymphomas, originating from the haematopoietic system, are seldom found in the heart due to the absence of a lymphoid system.^2^ Primary cardiac tumours are sporadic, with an incidence of 0.02% in a meta-analysis of 22 studies. The average life expectancy in a study was 2.2 years.^3^ Primary cardiac lymphomas constitute <1% of all extranodal lymphomas.^4^ Cardiac lymphomas may manifest in different ways, including dyspnoea, heart failure, pericardial effusion, arrhythmia, or may even be asymptomatic.^5^ Handling cardiac tumours is a complex task, especially when malignancy is a concern. The selection and early administration of diagnostic tests for critically ill patients are crucial. Given the vague symptoms, it might be challenging to diagnose these patients. The diagnostic management of cardiac tumours involves multiple approaches. Depending on tumour location, clinical presentation, and imaging data, diagnostic test selection may include transthoracic echocardiography, cardiac magnetic resonance imaging (MRI), and positron emission tomography.^6^

In this document, we delineate the diagnostic approach for cardiac diffuse large B-cell lymphoma in a patient who arrived at the emergency department exhibiting wide QRS tachycardia and dyspnoea. We also outline the effective diagnostic and treatment management of the patient, progressing from initial diagnosis to R-CHOP (rituximab-cyclophosphamide, doxorubicin, vincristine, and prednisone) chemotherapy, within the pertinent context of the literature.

## Summary figure

**Figure:**
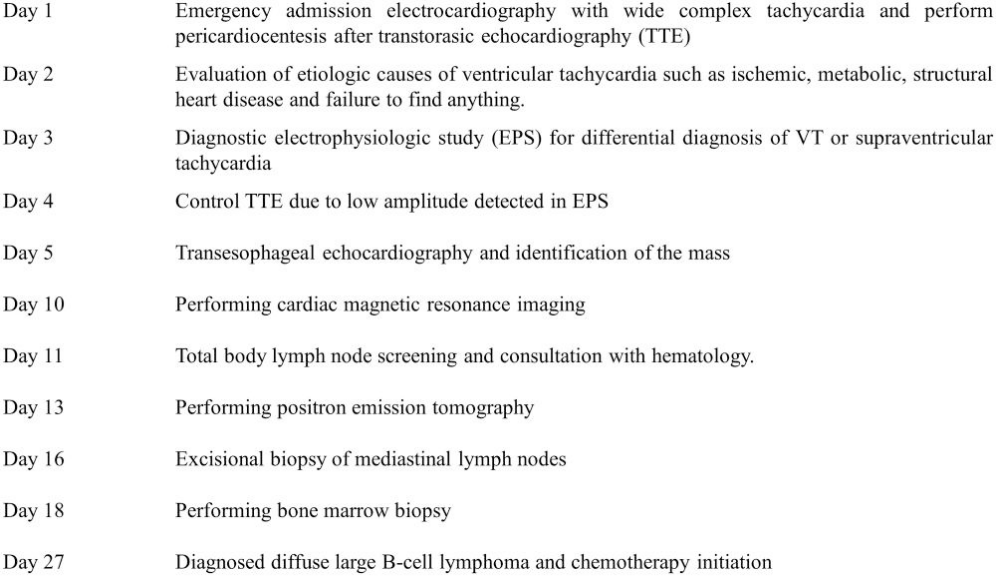


## Case

**Figure 1:**
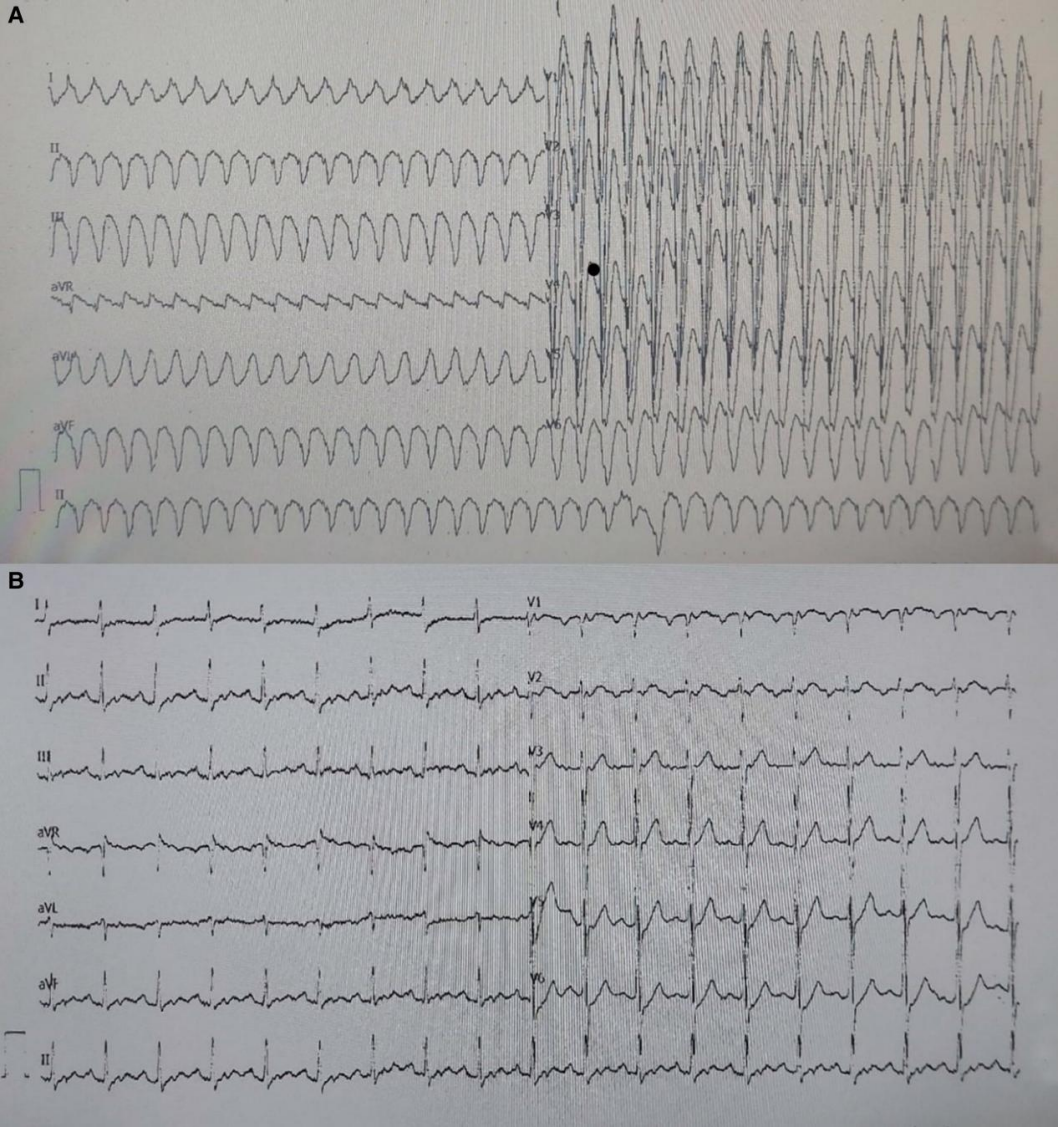
(*A*) Admission electrocardiogram with wide QRS tachycardia. (*B*) Control electrocardiogram after amiodarone administration. Sinus tachycardia. QRS duration 90 ms, PR interval 192 ms, and corrected QT interval 443 ms.

A 46-year-old male patient was admitted to the emergency department of our tertiary care hospital, presenting with dyspnoea and palpitations. The patient’s electrocardiogram showed tachycardia characterized by a wide QRS complex, with a heart rate of 234 beats per minute. An intravenous dose of amiodarone was administered promptly (*Figure 1A*) (a loading dose of 300 mg of amiodarone was given through intravenous infusion over 30 min. Amiodarone is the only intravenous antiarrhythmic drug available in our region, whereas procainamide is not available). After administering amiodarone, the follow-up electrocardiogram displayed a sinus rhythm of 110 beats per minute, with no ST abnormalities or electrical alternans (*Figure 1B*). After a thorough patient history assessment, the patient reported experiencing shortness of breath for the past week, which had intensified in the last hour and was accompanied by palpitations. The patient’s extensive medical history did not indicate any episodes of fever, weight loss, or abdominal pain. Five years earlier, the patient underwent coronary angiography due to an acute coronary syndrome, resulting in the implantation of a stent in the right coronary artery. The initial physical examination revealed a blood pressure of 118/70 mmHg and an oxygen saturation of 92%. The patient exhibited tachypnoea with a respiratory rate of 31 breaths per minute. Diminished S1 and S2 heart sounds were noted during cardiac auscultation, with no additional sounds. There was no evidence of pretibial oedema or hepatomegaly. However, the physical examination indicated jugular venous fullness. Upon lung auscultation, both lungs moved equally during respiration, and no rales or rhonchi were detected.

The patient’s laboratory results showed no abnormal parameters except for an elevated NT-proBNP value of 2722 ng/L (with a standard value below 300 ng/L). The haemograms were within the normal range, including white blood cell, lymphocyte, neutrophil, and platelet counts. The serum electrolytes, liver function, and renal function tests were also within normal limits.

**Figure 2:**
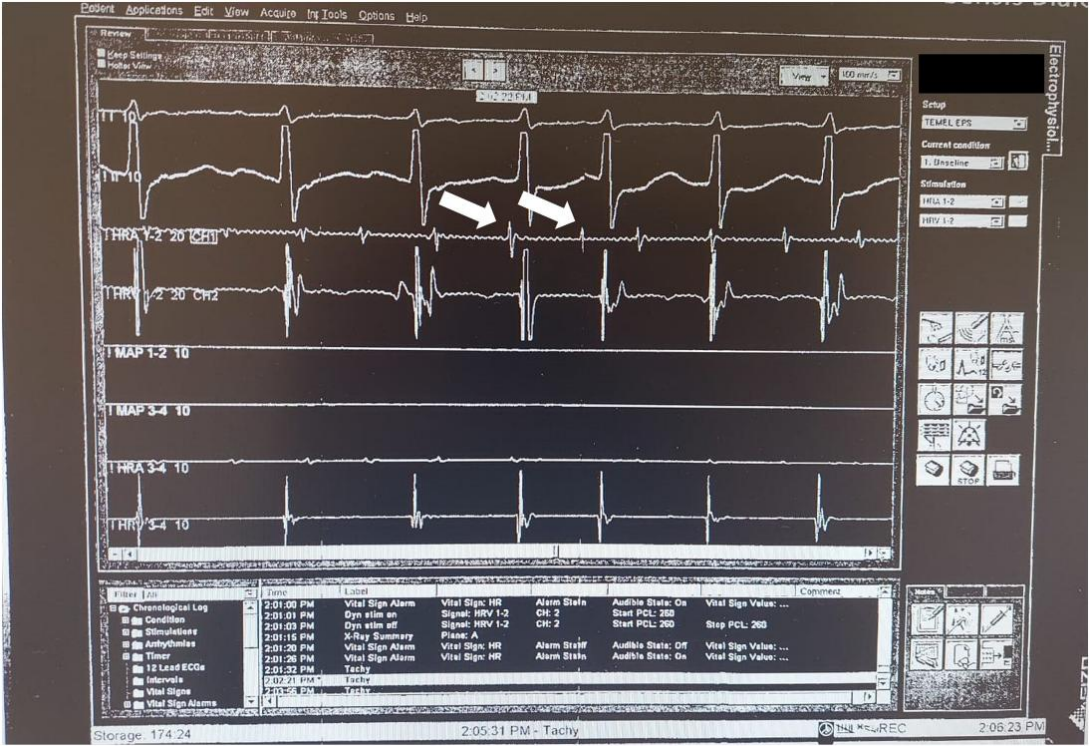
A low-amplitude signal from the lead in the right atrium during the electrophysiologic study (white arrows).

The emergency transthoracic echocardiogram (TTE) revealed a left ventricular ejection fraction of 60% with no regional wall motion abnormalities. A pericardial effusion measuring 2.4 cm at its widest point was observed surrounding the heart adjacent to the right ventricle. No reciprocal variation was noted in the mitral and tricuspid valves with diastolic collapse. Although the patient did not exhibit signs of tamponade syndrome, pericardiocentesis was performed due to the patient’s tachycardia and tachypnoea. Following the procedure, the patient’s tachycardia and tachypnoea improved. No diagnostic features were identified on the chest X-ray. The pericardial fluid was haemorrhagic and classified as exudative upon analysis. Upon reviewing the patient’s admission ECG, the absence of fusion and escape beats and an initial R wave duration shorter than 40 ms in aVR were evaluated as findings suggestive of SVT. The absence of RS in all precordial leads and the QS wave morphology in V6 on the admission ECG suggested VT. In this case, an electrophysiology study was planned to clarify the diagnosis. While awaiting the cytology results of the pericardial effusion, the patient was scheduled for a diagnostic electrophysiological study due to the presence of wide QRS tachycardia. During the electrophysiological study, it was noted that the signal amplitude recorded through the lead positioned in the right atrium was low, making successful atrial pacing impossible (*Figure 2*). Following the induction of the patient’s tachycardia via a catheter placed in the coronary sinus, high-transition atrial tachycardia with aberrant conduction was detected. Due to the patient’s weak right atrial signal and pacing difficulties, echocardiography and cardiac MRI were planned to rule out pathologies affecting the right atrial tissue. The follow-up echocardiography showed completely normal right ventricular function. Subsequently, the patient was scheduled for transoesophageal echocardiography.

**Figure 3:**
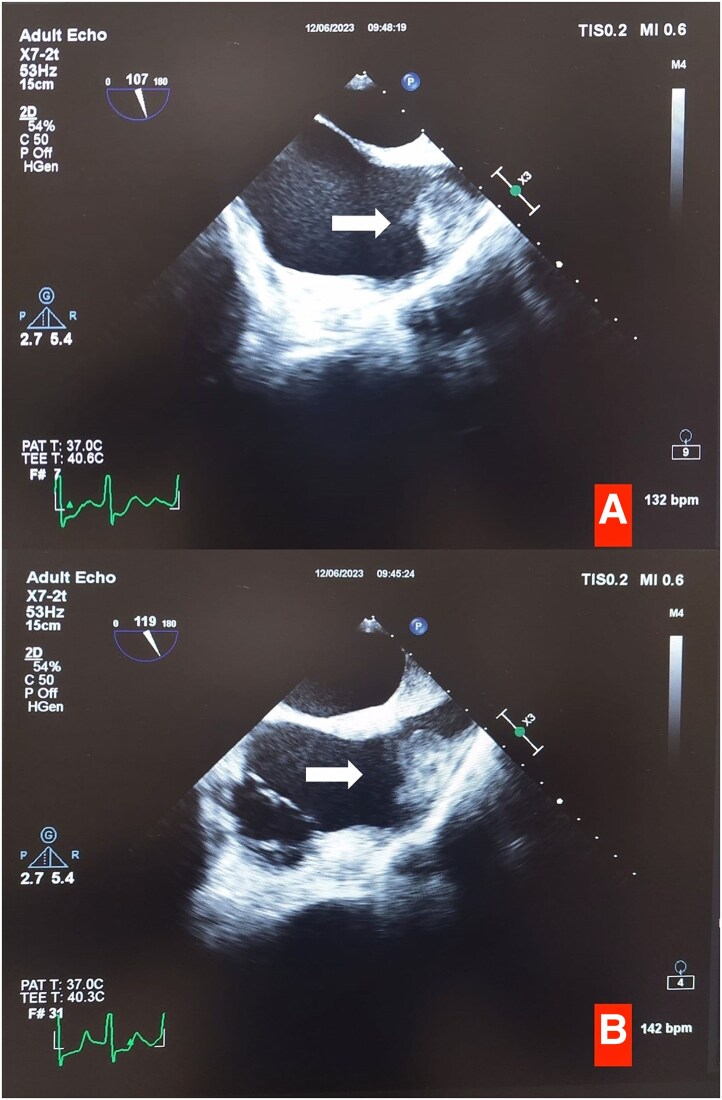
(*A*) Appearance of a mass extending from the right atrium to the right ventricular free wall in transoesophageal echocardiography (white arrows). (*B*) Mass extending from the right atrium to the superior vena cava in bicaval view in transoesophageal echocardiography (white arrows).

Transoesophageal echocardiography revealed masses with heterogeneous echogenicity extending from the superior vena cava to the right atrium (*Figure 3A*). We also noted a hyperechoic formation that we could not distinguish as either a mass or a thrombus, extending from the right atrium to the right ventricle and measuring 70 × 35 mm in diameter (*Figure 3B*, Supplementary material online, *Video S1*). Meanwhile, the pathological examination of the pericardial fluid indicated large lymphoid cells containing B cells, as shown by positive immunocytochemical staining for CD20.

**Figure 4:**
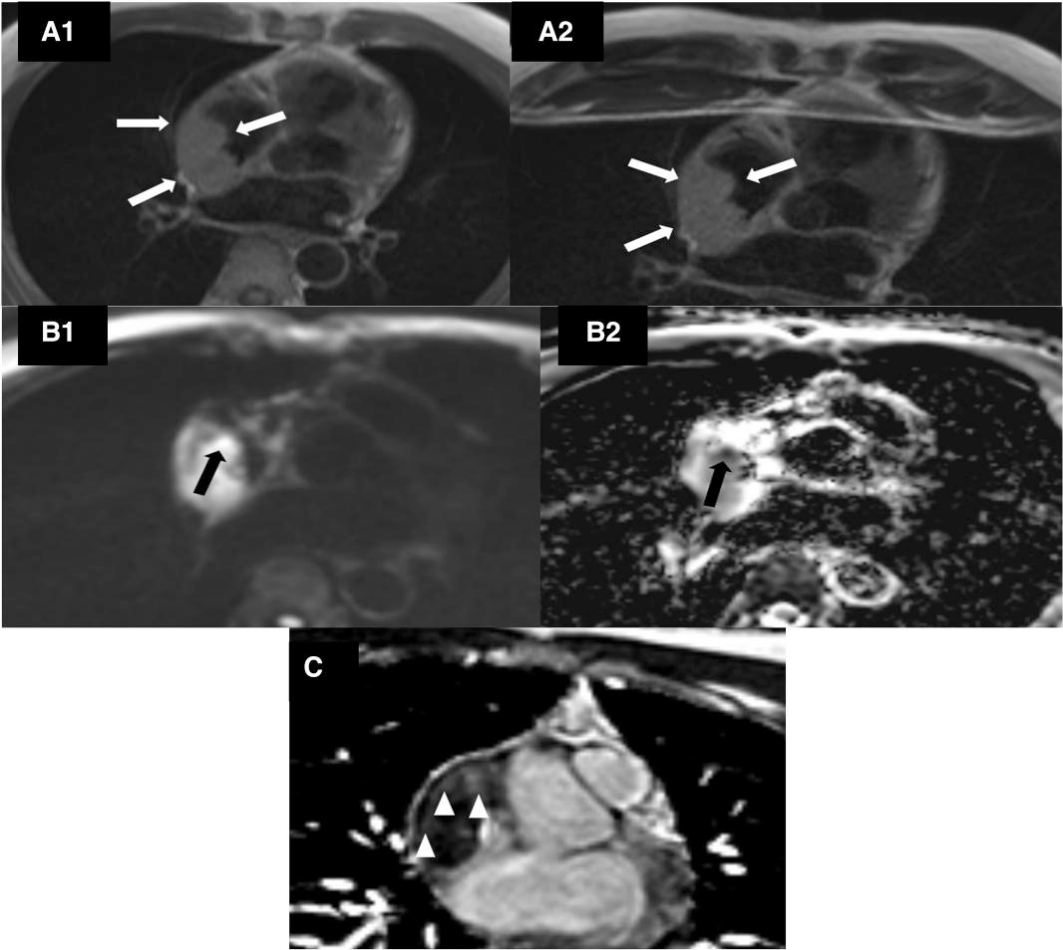
(*A1* and *A2*) Cardiac magnetic resonance imaging, T1 and T2-weighted TSE sequences, the mass is homogeneous and slightly hyperintense compared to the muscle (white arrows). (*B1* and *B2*) In the diffusion and ADC map with trace b = 1000 s/mm^2^, it is seen that some regions within the mass are markedly diffusion restricted (black arrow). The lowest ADC value was ∼550 × 10 −6 mm²/s. (*C*) Transverse plane ‘phase-sensitive inversion recovery’ sequence showing mild late gadolinium enhancement in the mass (arrowheads).

Cardiac MRI revealed a mass lesion measuring ∼63 × 30 × 79 mm, extending from the right atrium to the superior vena cava and the right atrial appendage. It infiltrated the free wall of the right ventricle, the pericardium, and the right atrial wall, showing distinct diffusion restriction in some areas (*Figure 4*). The lesion notably constricted the lumen of the superior vena cava at its entry point to the atrium (*Figure 4*). The patient’s cardiac MRI raised suspicion of cardiac lymphoma.

The patient’s PET-CT scan showed a dense cluster of hypermetabolic lymph nodes in the mediastinum and a hypermetabolic mass extending (See Supplementary material online, *Figure S1*) from the right atrium towards the superior vena cava. To assess for bone marrow invasion, a bone marrow aspiration was conducted. As a result, no bone marrow involvement was found.

**Figure 5:**
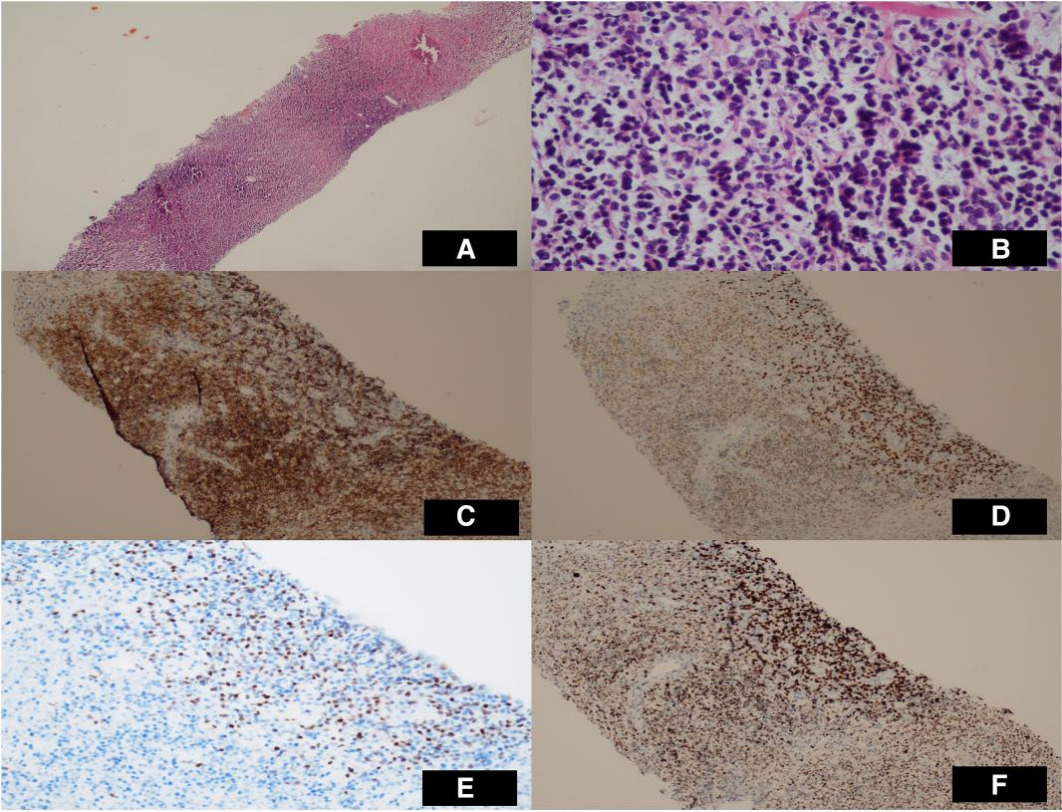
Histology specimens from mediastinal lymph node. (*A*) Atypical lymphoid infiltration in sections, H&E ×4. (*B*) Atypical cells with prominent nucleoli, H&E X. (*C*) CD20 immunohistochemical stain, ×10. (*D*) Positive staining with MUM1 immunohistochemical stain, ×10. (*E*) High proliferation with ki-67, ×20. (*F*) cMYC immunohistochemical stain, ×10.

In the histopathological examination of the excisional lymph node taken from the mediastinal lymph nodes, staining with CD20 and PAX-5 was observed. Internal control staining with CD10 was not observed. Positive staining with MUM1, negative staining with BCL 6 and CD30, and positive staining with BCL 2 were observed (*Figure 5*). The biopsy result was reported as diffuse large B-cell lymphoma. After diagnosing diffuse large B-cell lymphoma, the patient was referred to a haematology clinic and started on R-CHOP chemotherapy. The patient underwent six cycles of combination chemotherapy (R-CHOP: rituximab, cyclophosphamide, anthracycline, vincristine, and prednisone. The patient is currently in their sixth month of follow-up, with chemotherapy treatment ongoing (see Supplementary material online, *Video S2*: 6th month control TTE video).

## Discussion

Primary cardiac lymphoma tumours are rare, and the current literature is limited to only a few case reports due to their infrequent occurrence. The diagnostic process associated with our case was somewhat atypical; notwithstanding the presentation of wide QRS tachycardia, the patient did not manifest any of the B symptoms, including night sweats, weight loss, or fever. Endomyocardial biopsy is the gold standard for diagnosis; however, its invasive nature carries several associated risks. Additionally, this method is constrained by the necessity of accessing an experienced pathologist to interpret the obtained sample.^7,8^ All these factors increase the significance of advanced imaging modalities.

Cardiac lymphomas are often metastatic. The most common types of metastatic cardiac lymphomas include diffuse large B-cell lymphoma, T-cell lymphoblastic lymphoma, and Hodgkin lymphoma.^9^ Since primary cardiac lymphoma is a rapidly progressive and deadly disease, maintaining a high level of clinical suspicion and ensuring prompt diagnosis are essential. Cardiac lymphomas can easily go unnoticed if not suspected, as their symptoms are mild and non-specific. Due to delayed diagnosis and the lack of a standardized treatment approach, the prognosis is poor, with survival often lasting less than a month. However, with early diagnosis, some cases have shown survival extending up to 5 years.^10,11^ The treatment for primary cardiac lymphoma is similar to that of other non-Hodgkin lymphoma subtypes, typically involving six cycles of R-CHOP therapy.^12^

As in our case, lymphoma may manifest as a cardiac mass; furthermore, a mass measuring over 7 cm might not be discernible through transthoracic echocardiography. Incorporating cardiac MRI into the diagnostic process is essential for identifying intracardiac masses, which can accelerate diagnosis and the commencement of life-saving treatment. Our case also highlights the significance of utilizing cardiac MRI. Cardiac MRI is the preferred imaging modality for assessing cardiac tumours due to its excellent tissue characterization, high temporal resolution, multi-planar imaging capabilities, ability to localize the tumour accurately, and influence on cardiac function. Therefore, cardiac MRI plays a fundamental role in imaging and early diagnosis.^13^ This case highlights the significance of cardiac MRI in diagnosing elusive cardiac masses. Given the high mortality rates associated with cardiac masses in primary diseases, swift diagnosis is crucial.

Primary cardiac lymphoma may result in complications that are distinct from those observed in the case we present. Should the tumour be confined to the right ventricular outflow tract (RVOT) and either wholly or partially obstruct the outflow tract, the prognosis may be more threatening. In cases of RVOT obstruction, surgical intervention, despite its potential, does not enhance the prognosis.^14^ The initial course of chemotherapy should be administered cautiously, considering the risk of cardiac rupture during rapid tumour regression.^15^ The complications of primary cardiac lymphoma include decompensated heart failure, lymphoma progression, sepsis, arrhythmias, and sudden cardiac death^13^

## Conclusion

The prompt diagnosis and subsequent treatment of primary cardiac lymphoma are of paramount importance. Our objective was to enhance the existing literature by raising awareness regarding this disease and underscoring the importance of multimodal imaging techniques.

## Supplementary Material

ytaf065_Supplementary_Data

## Data Availability

The authors declare that data supporting the findings of this study are available within the article.

## Lead author biography

Dr Ömer Şahin continues his clinical studies in the Cardiology Clinic at Kayseri City Training and Research Hospital.

## Notes

**Consent:** The authors certify that they have obtained all appropriate patient consent forms. In the form, the patient has given his/her consent for his/her images and other clinical information to be reported in the journal. The patient understand that their names and initials will not be published and due efforts will be made to conceal their identity, but anonymity cannot be guaranteed. Informed consent was obtained from the patient’s family for the publication of this case report and any accompanying images.

**Funding:** The authors declare that they have not received any financial support for this article..
