# Supplementary figures and images for: Primary cardiac lymphoma presenting with wide QRS tachycardia: a case report
Source: Eur Heart J Case Rep. 2025 Mar 11;9(3):ytaf065. doi: 10.1093/ehjcr/ytaf065 (PMC11894249; doi:10.1093/ehjcr/ytaf065)

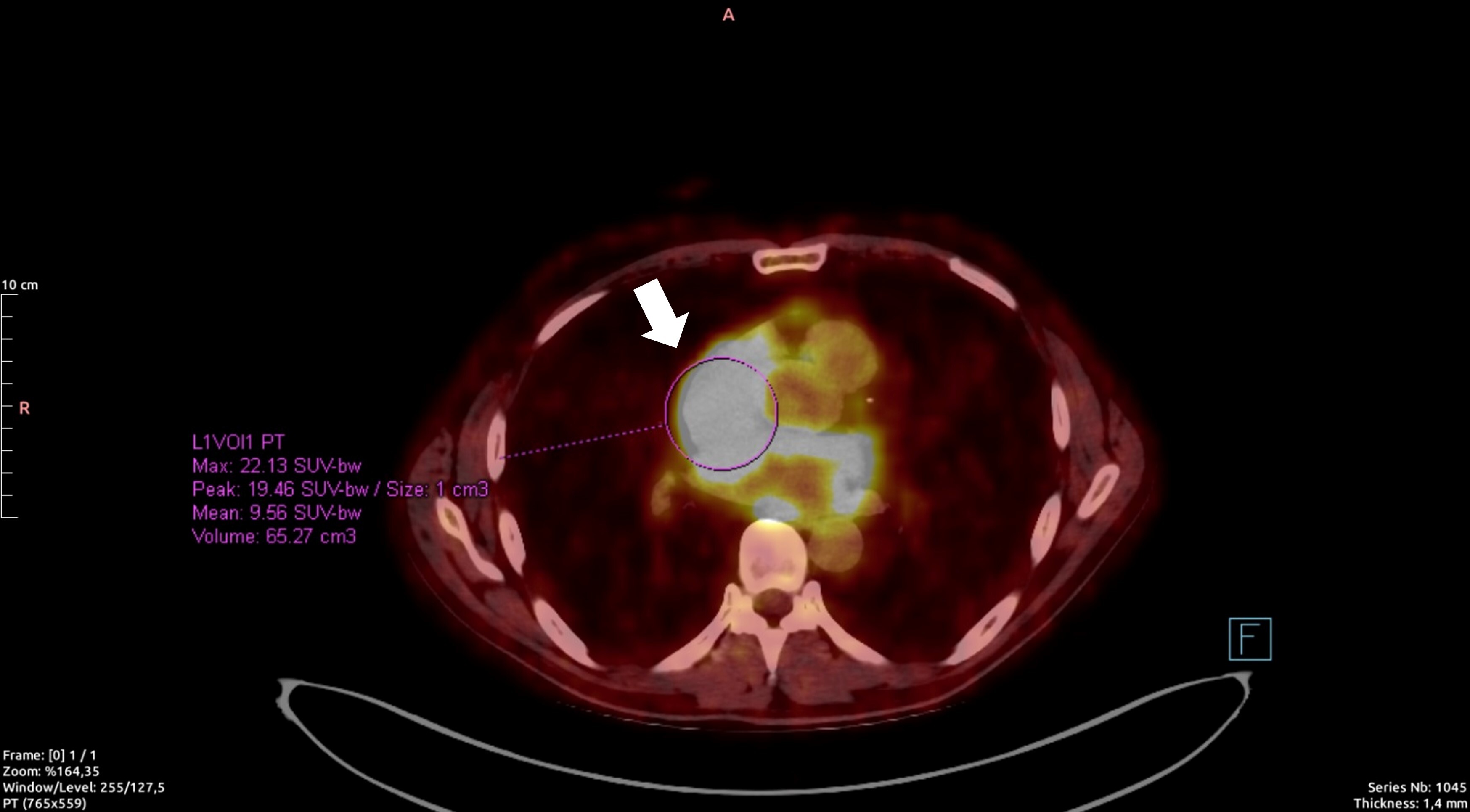

Supplement: ytaf065_Supplementary_Data [file ytaf065_supplementary_data.zip › supplementary. (1)-1737826219931.jpg]
